# Diversity of DNA viruses in the atmosphere of sub-Antarctic South Georgia

**DOI:** 10.3389/fmicb.2025.1726848

**Published:** 2026-01-28

**Authors:** Ritam Das, Lucie Malard, David A. Pearce, Peter Convey, Janina Rahlff

**Affiliations:** 1 Aero-Aquatic Virus Research Group, Faculty of Mathematics and Computer Science, Friedrich Schiller University Jena, Jena, Germany; 2 International Max Planck Research School for Biology and Computation (IMPRS-BAC), Max Planck Institute for Molecular Genetics, Berlin, Germany; 3 Department of Biology, Chemistry, Pharmacy, Freie Universität Berlin, Berlin, Germany; 4 Department F.-A. Forel for Environmental and Aquatic Sciences, University of Geneva, Geneva, Switzerland; 5 Department of Ecology and Evolution, University of Lausanne, Lausanne, Switzerland; 6 School of Geography and Natural Sciences, Faculty of Science and Environment, Northumbria University, Newcastle upon Tyne, United Kingdom; 7 British Antarctic Survey, Natural Environment Research Council, Cambridge, United Kingdom; 8 Department of Applied Sciences, Zoology, University of Johannesburg, Auckland Park, South Africa; 9 Millennium Institute – Biodiversity of Antarctic and Sub-Antarctic Ecosystems (BASE), Santiago, Chile; 10 Cape Horn International Center, Puerto Williams, Chile; 11 School of Biosciences, University of Birmingham, Edgbaston, United Kingdom; 12 Leibniz Institute on Aging – Fritz Lipmann Institute (FLI), Jena, Germany; 13 European Virus Bioinformatics Center, Jena, Germany; 14 Centre for Ecology and Evolution in Microbial Model Systems (EEMiS), Department of Biology and Environmental Science, Linnaeus University, Kalmar, Sweden

**Keywords:** aerosol, Coriolis, marine, phage, sea, virome, airborne, metagenomics

## Abstract

Studying airborne viruses in remote environments like the sub-Antarctic island of South Georgia offers key insights into viral ecology, diversity, and their role in shaping ecosystems through microbial and nutrient interactions. We analyzed airborne viral community composition at two sites in South Georgia. Sampling took place using multiple methodologies, with the data produced subjected to viral metagenomics. The Coriolis *μ* device (wet collection) was the most effective, yielding 30 viral scaffolds. Two-thirds of the scaffolds were only obtained from the coastal location, indicating that location influences airborne viral diversity. Protein-based clustering of 39 viral operational taxonomic units (vOTUs) revealed similarities of 15 with known marine viruses, suggesting oceanic influence on the airborne viral community. Protein homologs related to UV damage protection and photosynthesis from two airborne vOTUs were widely distributed across major oceans, suggesting their potential role in supporting the resilience of marine microorganisms under changing climate conditions. Some vOTUs had protein similarities to viruses infecting extremophiles, indicating viral adaptations to harsh environments. This study provides a baseline for understanding the complexity and sustainability of airborne viral communities in remote ecosystems. It underscores the need for continued monitoring to assess how these communities respond to shifting atmospheric and ecological conditions.

## 1 Introduction

The cryosphere, encompassing ice sheets, glaciers, permafrost, and sea ice, covers approximately 20% of Earth’s surface, and hosts some of the planet’s most climatically sensitive ecosystems (Malard et al., 2019). Characterized by chronically low temperatures and harsh environmental conditions and covered by continental-scale ice sheets on average 2 km thick, the world’s fifth-largest continent, Antarctica, is a critical driver of global climate and ocean systems. Its final separation from southern South America and Australia around 33 million years ago in the final stages of the breakup of Gondwana was followed by cooling and the eventual formation of its ice sheets, and led to the evolution of a diverse cold-adapted microbiota (Pearce et al., 2009). Aerial dispersal is a major route for the invasion of new species (Pretorius et al., 2023), particularly those that are small enough, or have suitable propagules, to be uplifted into and transported into the air column. Antarctica is increasingly appreciated to host a complex microbial diversity (Cary et al., 2010; Cavicchioli, 2015), much of which appears to be endemic to the continent (Vyverman et al., 2010; Verleyen et al., 2021). However, few studies have yet investigated airborne bacteria in the Antarctic region (Pearce et al., 2010; King-Miaow et al., 2019; Cao et al., 2021; Malard et al., 2022). Furthermore, there is a notable scarcity of research on both Antarctic viral diversity generally and on airborne viruses specifically (reviewed by Heinrichs et al., 2024), which is rather astonishing considering that these biological entities are ubiquitous and reach global numbers of 10^31^ viral particles (Hendrix et al., 1999). One reason for the absence of viral investigations is that DNA yields from aerosols are typically low, due to the extremely low biomass, with ambient viral particle concentrations estimated at 3 × 10^4^ m^−3^ (Després et al., 2012), rendering airborne viruses understudied components of outdoor environments, whose detection is technically challenging (Prussin et al., 2014; Tastassa et al., 2024). Instead, 16S rRNA gene surveys (metabarcoding) involving a PCR step for amplification are often applied (Nga et al., 2024; Vishwakarma et al., 2024), which are appropriate for studies of prokaryotes. Viruses typically do not disperse in the air as free particles; instead, they often attach to soil dust or marine organic aggregates, which can then form aerosols (Reche et al., 2018). The deposition rates of viruses in the atmosphere, which can be up to ~460 times higher than those of bacteria, are positively correlated with organic aerosols (Reche et al., 2018). This suggests that viruses may have longer residence times in the atmosphere than microbes or other microscopic propagules, likely due to their smaller size (Alsante et al., 2021). Consequently, there is greater likelihood of their dispersal over long distances.

Sea spray aerosols (SSA), which are formed by the primary emission from the ocean, are the key component by mass of marine aerosols and play a crucial role in the Earth’s climate system (Quinn et al., 2015). A virus infecting the marine coccolithophore *Emiliania huxleyi* can lead to their rapid demise and induce coccolith shedding, the process by which these marine algae release their calcium carbonate plates into the surrounding water. This can lead to subsequent incorporation of the calcite units (coccoliths) to the SSA, with these viruses acting as a regulator of the mass of marine aerosols (Trainic et al., 2018). Through aerosol-mediated long-distance transport, viruses could constitute an ecological and evolutionary driving force beyond marine ecosystems alone, for instance through rain-washout events, where aerosolized particles are removed from the atmosphere by precipitation, or through direct sedimentation (Reche et al., 2018). Such events would allow airborne viruses to interact with microbes and other organisms in terrestrial ecosystems, affecting their abundance, community composition and distribution in the recipient ecosystems (Reche et al., 2018).

Investigations of viral diversity and lifestyle have been conducted on microbial viruses from Antarctica’s lakes (Kepner et al., 1998), sea ice (Paterson and Laybourn-Parry, 2011; Luhtanen et al., 2018), under the ice shelf (Lopez-Simon et al., 2023), soils (Zablocki et al., 2014), from cryoconite holes (Sommers et al., 2019) and marine ecosystems including the Southern Ocean (Guixa-Boixereu et al., 2002; Liang et al., 2015). In a study of Antarctic surface snow, similar viral populations were described from locations separated by several hundred to thousands of kilometers and overlaps between snow and marine viral populations were apparent (Rahlff et al., 2022). Flavobacteria from Antarctic snow carry adaptive immunity in the form of clustered regularly interspaced short palindromic repeats (CRISPR)-Cas systems, indicating that defense mechanisms against mobile genetic elements are present (Lopatina et al., 2016). Recent work has demonstrated that viral diversity in the Southern Ocean around the Western Antarctic Peninsula is complex and has uncovered both diversity and novelty amongst the viruses present. As well as tailed dsDNA bacteriophages representing the class Caudoviricetes, abundant eukaryotic viruses including polinton-like viruses, nucleocytoplasmic large DNA viruses (NCLDV), and virophages have been reported (Piedade et al., 2024). A study in the Antarctic and sub-Antarctic region of South Georgia observed the mortality and morbidity of several key indigenous species due to the spread of a high pathogenicity avian influenza virus H5N1 (Banyard et al., 2024). Therefore, monitoring viruses in isolated (including anthropogenic) locations could help track changes in viral communities over time, determine their stability and resilience, and assess the importance of anthropogenic influence. Furthermore, contemporary increase in global temperature and its regional impact on cryosphere-associated life forms presents a unique opportunity to understand ecosystem function, which could help shape a sustainable ‘One World’ for the future.

In this study, we used viral metagenomics to investigate the spatio-temporal variability of airborne viruses on the remote sub-Antarctic island of South Georgia in the South Atlantic Ocean, collected over 7 days using different air samplers and a rain collector. We hypothesized that the surrounding ocean would be a major contributing source for atmospheric viruses in the bioaerosols but also sought homologs of viral proteins obtained in our South Georgia samples in datasets available from other oceans to which viruses are probably dispersed or from which they are recruited.

## 2 Materials and methods

### 2.1 Field sampling and filtration

**Figure 1:**
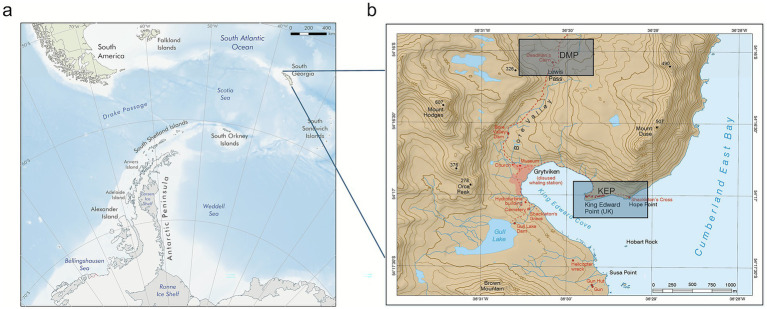
Map showing the location of South Georgia **(a)** and the two sampling sites DMP = Deadman’s Cairn, Lewis Pass, and KEP = King Edward Point **(b)**. Sources: **(a)** Mapping and Geographic Information Centre, British Antarctic Survey, 2025; bathymetry: Weatherall et al. (2024), coastline: SCAR Antarctic Digital Database, 2024 for areas south of 60°S and Natural Earth for areas north of 60°S **(b)** “South Georgia and The Shackleton Crossing” by the British Antarctic Survey, 2021, 1:200,000 and 1:40,000 scale, BAS (Misc 12), Cambridge, British Antarctic Survey. The sampling locations in 1b were previously presented in Das et al. (2024) and later also included in a different manuscript (Malard et al., 2025).

Air and rain samples were collected in South Georgia (latitude −54.4, longitude −36.5), which is ~1800 km from Antarctica to the south, ~1,400 km from the tip of the Antarctic Peninsula to the south-west and ~2,150 km from southern South America to the west. The collections took place from October 15 to 21, 2021, at two different sampling sites: King Edward Point (KEP) and Deadman’s Cairn at Lewis Pass (DMP) (Figure 1). These sites represent two different sampling altitudes of ~3 and ~200 m above sea level, respectively. Site KEP (−54.283528, −36.495194) was directly at the coast, while DMP was located 2 km inland (−54.269964, −36.503672, Figure 1) (Malard et al., 2025). Three different air samplers were used, namely the “wet” Coriolis® *μ* air sampler, “dry” Coriolis Compact air sampler (both Bertin Technologies SAS, Montigny-le-Bretonneux, France), and a Whatman Vacuum Pump attached to a Sartorius 250 mL filter holder collector (“pump”). The samplers were positioned about 1 m above the ground surface. For rain sampling, a plastic funnel attached to a 1 L sterile collection bottle (“collector”) was used (at KEP only). At the DMP site, only the wet and dry Coriolis samplers could be used as they function on batteries and do not require main power. Sampling duration was 1–6 h when using the Coriolis instruments with high flow rates, depending on weather conditions, and extended up to 22 h when air was collected using the vacuum pump. Rain collectors were used for 15 h (Supplementary Table S1). We extracted hourly wind speed, wind direction, pressure, and air temperature data for the sampling period (Supplementary Table S1) from the Grytviken Automatic Weather Station. For each sample, the corresponding values of each variable were averaged to cover each sampling duration, allowing us to obtain average environmental conditions for each sample.

### 2.2 DNA extraction and sequencing

Cellulose nitrate membrane filters with air samples were sliced into 0.5 mm wide strips using a sterilized scalpel to maximize the filters’ exposure to the cell lysis buffer during DNA extraction. For samples collected in nuclease-free water using the Coriolis wet device, the collection cones were vortexed (SciQuip, Shropshire, UK) before being filtered through 0.2 μm pore size cellulose nitrate membrane filters (GE Healthcare Life Sciences, Chicago, IL, USA) using a Welch WOBL vacuum pump (Welch, Mt. Prospect, IL, USA) for 10 min. Samples obtained via the Coriolis Compact air sampler (the dry device) were resuspended in 15 mL of DNA/RNAase-free water, manually shaken, and vortexed before undergoing the same filtration process. All pre-processing was conducted in a Class II cabinet, and the vacuum pump, tubing, and filtration apparatus were sterilized with 70% ethanol before use. DNA extraction from air samples was performed using the Qiagen PowerSoil kit (Qiagen, Hilden, Germany) according to the manufacturer’s instructions. Sterile water controls taken during sampling and extraction kit controls, although not sequenced for metagenomics, showed no contamination in the 16S rRNA gene amplicon dataset, as detailed in a separate study (Malard et al., 2025). DNA extracts were submitted to NU-OMICS sequencing facility at Northumbria University, Newcastle, for Illumina NextSeq shotgun metagenomics sequencing. DNA libraries were prepared using the Nextera XT DNA Library Prep Kit, following the manufacturer’s instructions. Sequencing was conducted on an Illumina NextSeq system (Illumina Inc., USA) with V2.5 300-cycle chemistry.

### 2.3 Metagenomic analysis

Sequencing reads went through adapter trimming using BBduk within BBTools (Bushnell, 2014), and afterwards Sickle v.1.33 (Joshi and Fass, 2011) was run in paired-end mode and ‘-t sanger’ setting. Taxonomic profiling of microbes was performed using the tool mOTUs v.3.0.2 on trimmed reads with options ‘-A’ (reports full taxonomy), ‘-c’ (reports counts) ‘-M’ (to save intermediate marker gene cluster count) including a separate run to retrieve unassigned taxa. The tool mOTUs employs universal, protein-coding, and single-copy phylogenetic marker gene sequences (Milanese et al., 2019; Ruscheweyh et al., 2022). The trimmed reads were subsequently assembled using metaSPAdes v.3.15.5 (Nurk et al., 2017) and metaviral SPAdes (Antipov et al., 2020). Assemblies were combined, and viral scaffolds were predicted by running VIBRANT v.1.2.1 (Kieft et al., 2020), VirSorter v2 with setting ‘--include-groups “dsDNAphage,ssDNA, NCLDV,lavidaviridae”’ and default score (Guo et al., 2021), and geNomad v.1.3 (Camargo et al., 2024). Viral scaffolds detected by the different tools were combined and filtered to a minimum length of 3,000 bp. The numbers of assembled viral scaffolds were summed for each sampling device. As the number of viruses and the genome size were very low, assemblies across all read files (respective forward and reverse reads of all samples merged) were performed using the above tools and additionally MEGAHIT v.1.2.9 (Li et al., 2015). The resulting viral scaffolds were combined with the previous ones, and a size cut-off of 5,000 bp was applied. Viruses were clustered using VIRIDIC v.1.0 r3.6 (Moraru et al., 2020), and only one viral operational taxonomic unit (vOTU) of each species cluster (demarcation threshold = 95% intergenomic similarity) was used in downstream analysis, and CheckV v.1.0.1 (Nayfach et al., 2021) was run. To determine if a vOTU was present in a sample, reads were mapped back to vOTUs using Bowtie 2 v.2.3.5.1 (Langmead and Salzberg, 2012) with settings ‘--mp 1,1 --np 1 --rdg 0,1 --rfg 0,1 --score-minL,0,-0.1’ (Nilsson et al., 2020), which ensures that only reads with 90% sequence identity map to the vOTU. To follow guidelines of Roux et al. (2017), a vOTU was considered present if at least 75% of the scaffold was covered with reads, which was checked using the ‘calcopo.rb’ script (Rahlff et al., 2022). Mean coverages of vOTU were determined using the ‘04_01calc_coverage_v3.rb’ script (Bornemann et al., 2023), and coverages were normalized to sequencing depth. Relative abundance was calculated as the proportion of each normalized coverage relative to the total, expressed as a percentage. Gene calling was performed using Prodigal v.2.6.3 (Hyatt et al., 2010), and for viral clustering, vConTACT2 v.0.9.19 (Bolduc et al., 2017) was run on the vOTU proteins together with those from the 1August2023 viral reference database (https://github.com/RyanCook94/inphared/tree/a330daa635cd3c78843d470668cb22ff842960e4) derived from INPHARED (Cook et al., 2021). Results were compiled using graphanalyzer v.1.5.1 (Pandolfo et al., 2022), and a network was built in Cytoscape v.3.10.3 (Shannon et al., 2003). PhaMer (Shang et al., 2022) and PhaGCN (Shang et al., 2021) within PhaBOX v.1 (Shang et al., 2023) were used to discriminate phages from non-phages, and to determine the taxonomic rank of a phage at the family level, respectively. VirClust webtool (Moraru, 2023) with default settings was run on the airborne vOTUs. ViPTree v.4.0 (Nishimura et al., 2017) was run with settings for dsDNA for nucleic acid type of reference viruses and “Any host” as the host category. Further virophage classification was performed using https://github.com/simroux/ICTV_VirophageSG (Roux et al., 2023b). In a previous, non-polar study, viral genomes retrieved from aerosols and rainwater had a significantly higher guanine/cytosine (GC) base content compared to marine viruses (Rahlff et al., 2023), which is why the GC content of the Antarctic air vOTUs was further explored. The GC content of vOTUs was determined using EMBOSS v. 5.0.0 (Rice et al., 2000). Annotations of vOTUs were performed using DRAM-v v.1.5.0 (Shaffer et al., 2020), which includes annotations from the databases Pfam, VOGDB, KOfam, dbCAN, and RefSeq viral. The vOTUs were BLASTed against the IMG/VR Viral Nucleotide Database v.4 with an e-value cut-off of 1e-5 (Camargo et al., 2023). Domain analysis of the proteins was performed using the NCBI Conserved Domain Search website (Marchler-Bauer et al., 2015). A BLASTp analysis for selected amino acid sequences from two vOTUs was run against the Ocean Gene Atlas (Villar et al., 2018; Vernette et al., 2022) using the Tara Oceans Microbiome Reference Genome Catalog v1 OM-RGC_v1 (based on metagenomes) and an e-value cut-off of 1e-10. Photosynthetically active radiation (PAR) was chosen as the environmental variable based on which the spatial distribution and abundance (average copies per cell) of the protein homologs of vOTU_35_ORF14 and vOTU_39_ORF2 were explored, because the protein homologs are functionally related to PAR or ultraviolet radiation, which both correlate with global solar radiation (Escobedo et al., 2011). The phylogenetic tree acquired from the Ocean Gene Atlas was edited using the Interactive Tree of Life (iTOL) v.6 website (Letunic and Bork, 2024). Overlaps of vOTUs between stations were visualized with Venn Diagrams made with the Ugent webtool (https://bioinformatics.psb.ugent.be/webtools/Venn/). Host predictions were performed using iPHoP v.1.3.3 and the database Aug_2023_pub (Roux et al., 2023a). Metagenome-assembled genome (MAG) binning was performed using MaxBin2 v.2.2.7 (Wu et al., 2016) and MetaBAT2 (Kang et al., 2019), but resulted in only a few low-quality bins (<70% completeness and >10% contamination) as assessed by CheckM2 (Chklovski et al., 2023). These bins were excluded from further analysis.

### 2.4 Diversity analysis and statistics

Community and diversity analysis of vOTUs was performed using phyloseq v. 1.48.0 (McMurdie and Holmes, 2013) within the R programming environment v.4.4.0 (Team R.C, 2019). Plotting and statistical analysis of Shannon-Wiener index was performed in Graphpad Prism 10.

## 3 Results

### 3.1 Viral community composition is affected by sampling site, collection device, and daily fluctuations

**Figure 2:**
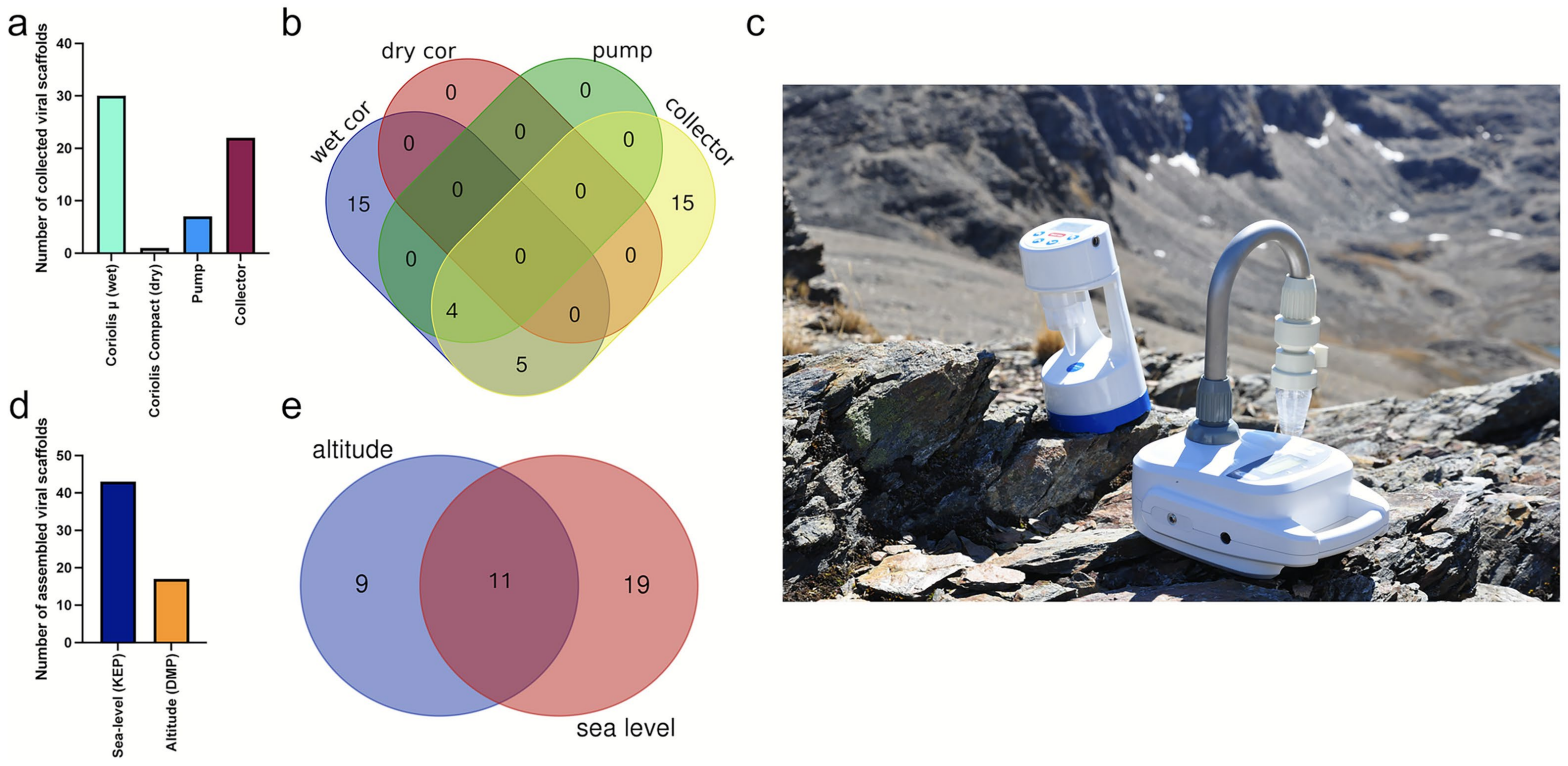
Airborne virus sampling with different collection devices from two sampling sites in South Georgia. **(a)** Number of collected viral scaffolds >3,000 bp per sampling device; for description of sampling devices, see main text. **(b)** Venn diagram showing overlapping vOTUs for different sampling devices based on read mapping. **(c)** Coriolis *μ* and Coriolis Compact sampler in the Antarctic landscape (Image: David Pearce). **(d)** Number of collected viral scaffolds >3 kb by sampling site, **(e)** Venn diagram showing overlapping vOTUs (>5 kb) between sampling sites based on read mapping.

The different sampling devices (Figure 2c) collected different numbers of viral genome fragments. From the 60 assembled vOTUs >3 kb length, most vOTUs (30) were assembled from samples collected with the wet Coriolis and only one vOTU from the dry Coriolis (Figure 2a). Seven and 22 vOTUs were assembled after collection with the vacuum pump and the rain collector, respectively. Of the 60 assembled vOTUs, 43 and 17 vOTUs were collected at the coastal KEP site and the inland higher altitude DMP site, respectively (Figure 2d). From these assembled scaffolds and the cross-assembled ones, 39 vOTUs >5 kb were identified and further analyzed. Mapping reads to these vOTUs showed that 15 unique vOTUs were present in samples taken with the rain collector, another unique 15 vOTUs in samples from the wet Coriolis, and 9 vOTUs occurred in samples obtained by both devices, suggesting that these two sampling methods complement each other for virus sampling (Figure 2b). Eleven vOTUs were shared between both sampling sites, while 19 were only present at KEP and 9 at DMP (Figure 2e).

**Figure 3:**
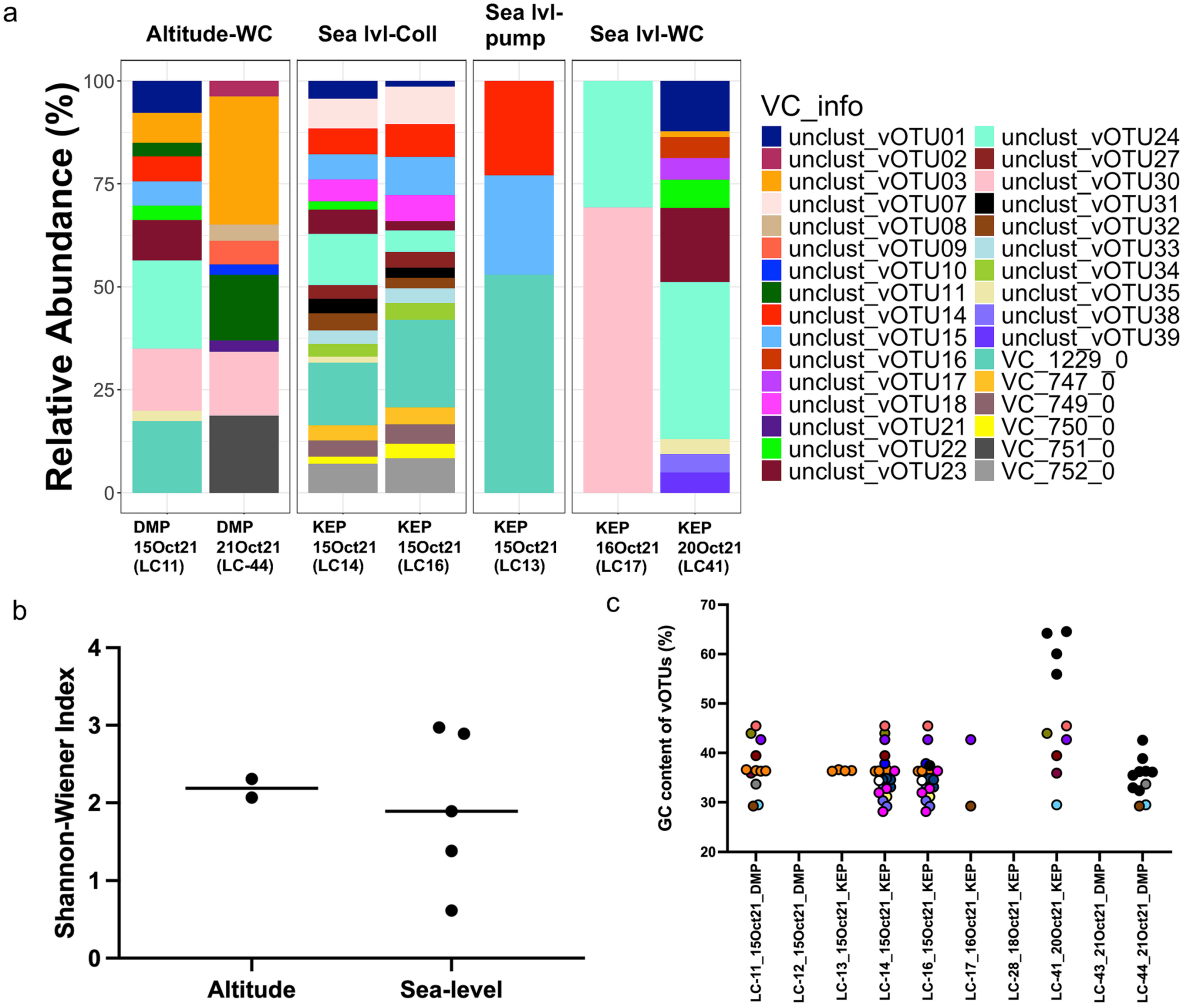
Viral cluster and vOTU distribution and relation to guanine/cytosine (GC) content. **(a)** Relative abundance of viral clusters (VC) and unclustered viral operational taxonomic units (vOTUs) across stations. **(b)** Shannon-Wiener index for viruses separated by study site. **(c)** GC content of vOTUs versus sampling date and site. If vOTUs were found in several samples based on read mapping, they have the same color; if they are unique for a sample, they are black. DMP = Deadman’s Pass, KEP = King Edward Point, Sea/lvl = Sea-level, WC = wet Coriolis, LC refers to internal sample identification.

The vOTUs were clustered into viral clusters (VCs) by vConTACT2, corresponding to genus-level clusters, while “singletons” and “outliers” may represent novel viruses. Relative abundance analysis showed that VC_1229_0 (containing vOTU_12 and vOTU_13), vOTU_24, and vOTU_30 were the most abundant (based on coverage depth) and prevalent (based on coverage breadth) VCs in the community (Figure 3a and Supplementary Table S2). Based on Virsorter2, VC_1229_0 might contain virophage vOTUs, family Lavidaviridae, which could, however, not be further validated by using virophage-specific markers. VOTU_30 was classified as Bamfordvirae by geNomad, which includes NCLDV. Shannon-Wiener diversity was highest for vOTUs sampled with the rain collector at KEP (2.9 and 3.0) compared to the wet Coriolis at DMP (2.1 and 2.3) and compared to the wet Coriolis and pump at KEP (0.6–1.9) and thus did not show a clear Shannon-Wiener diversity difference between the two sites but may indicate a joint effect of the sampling device and site on alpha-diversity (Figure 3b).

During sampling on October 15, 2021, both the vacuum pump and the collector captured some of the most abundant vOTUs (Figure 3a). Based on read mapping, vOTUs and VCs reoccurred and co-occurred on different days and at both sites, but also new vOTUs appeared (Figures 3a,c). Rain samples (LC-14 and LC-16) contained some vOTUs that were also recovered from air on October 15, suggesting that these overlapping vOTUs were washed out by falling rain from the atmosphere or resulted from raindrops that were collected with the aerosol sampler. Some VCs were only detected in the rainwater, namely VC_752_0, VC_750_0, VC_749_0 and VC_747_0 (all predicted Caudoviricetes). Notably, on one day (LC-41), where viruses were sampled at KEP with the wet Coriolis, four vOTUs present in the sample based on read mapping had a GC content of 61.8% ± 4.0 (mean ± standard deviation, Figure 3c and Supplementary Table S3).

### 3.2 Protein-based clustering reveals similarities to known marine viruses

**Figure 4:**
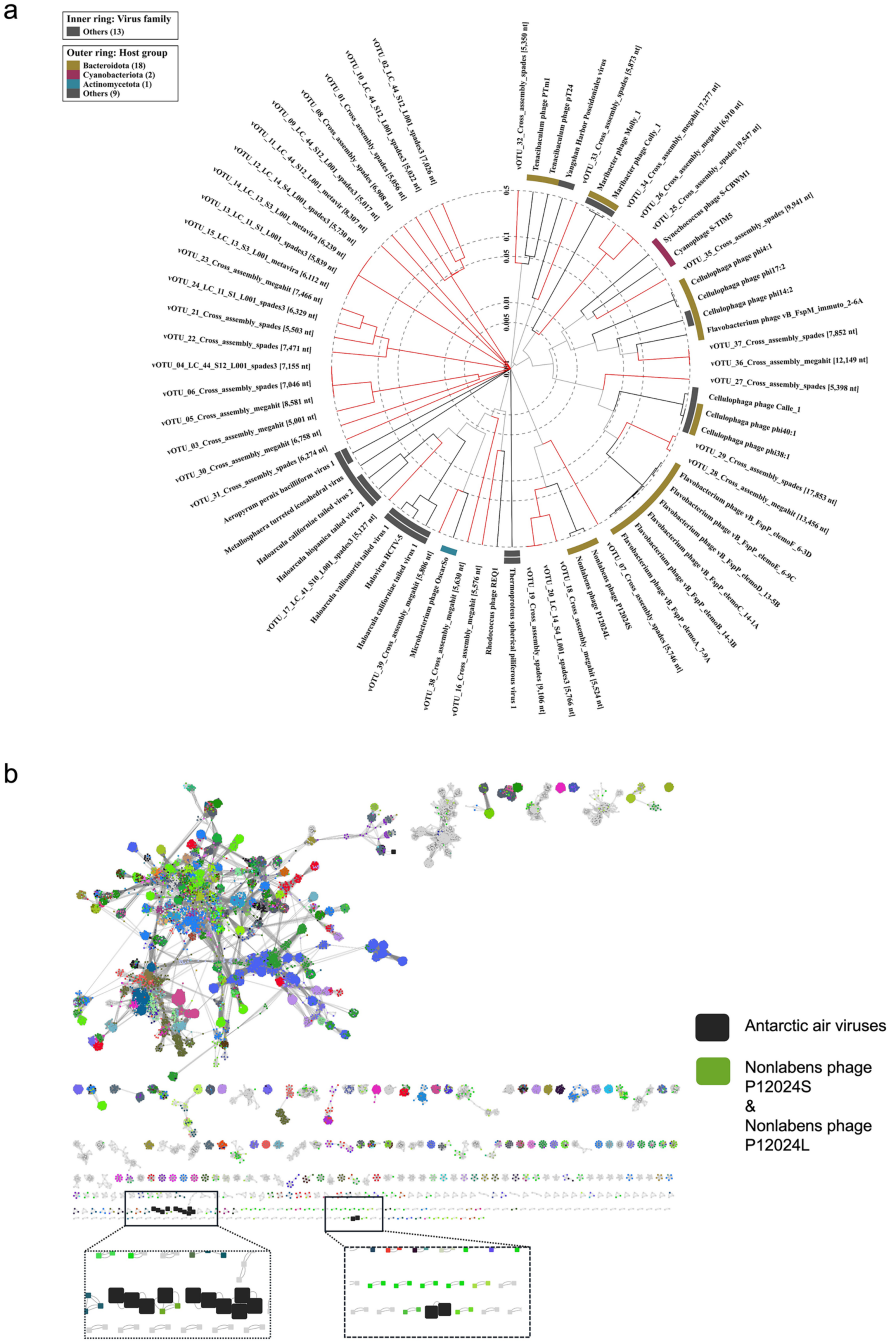
Viral protein-based clustering. **(a)** Proteomic tree of 39 viral operational taxonomic units (red branches) and their related viruses constructed using ViPTree. **(b)** Protein-sharing network of Antarctic vOTUs (big black squares) forming viral clusters with Nonlabens phage P12024S and P12024L and with each other.

According to the classification of the 39 vOTUs by geNomad, Adintoviridae (3), Caudoviricetes [15 including Crassvirales (3)], Imitervirales (1), Bamfordvirae (2), unclassified (10) or not determined by the tool (8) were found. VirSorter2 predicted dsDNA phages (13), Lavidaviridae (9), NCLDV (7) and not determined (10) (Supplementary Table S3). Depending on the virus identification tool, evidence for the presence of 13–17 phages among the 39 vOTUs was found (Figures 4a,b and Supplementary Table S3). Four phages were predicted to be family Demerecviridae and two others family Straboviridae (Supplementary Table S3).

**Figure 5:**
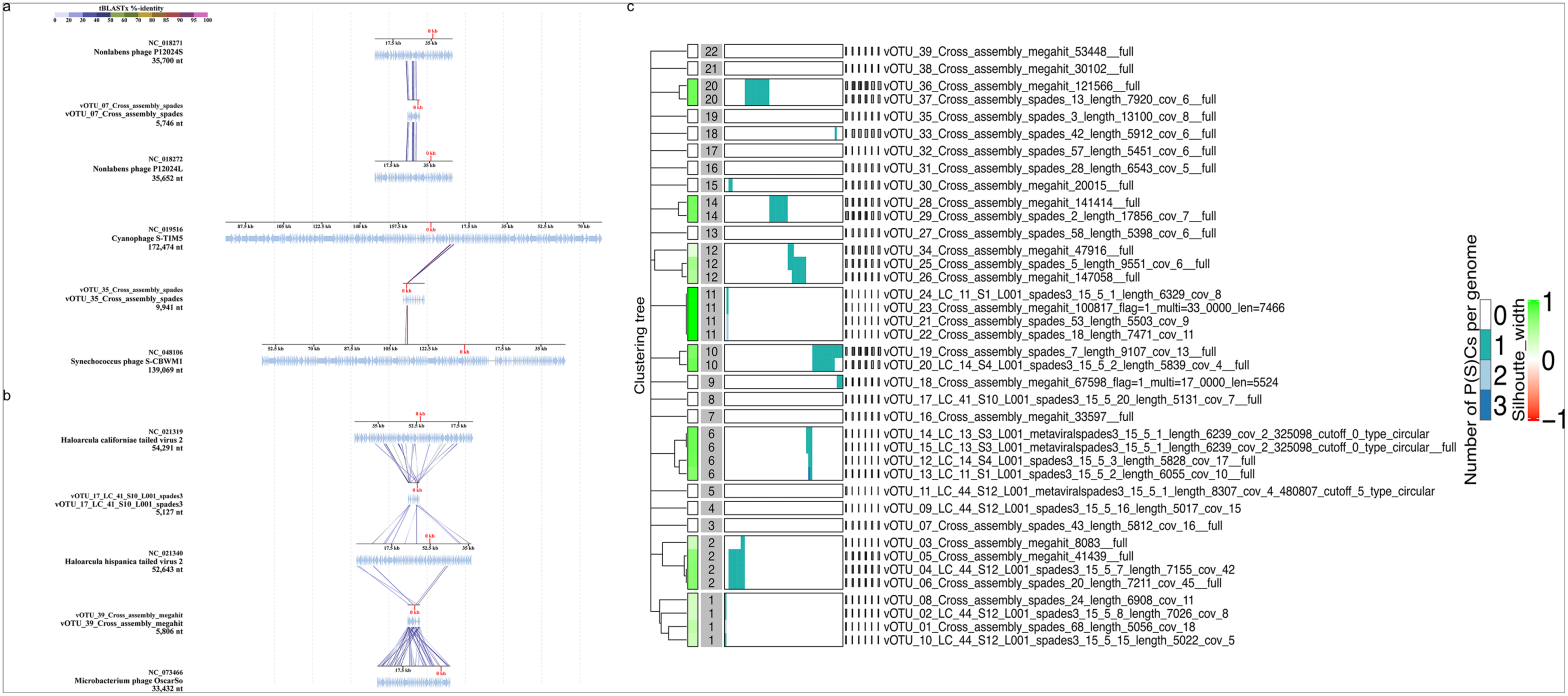
Sequence alignment and proteome clustering of annotated vOTUs. **(a)** Sequence alignment of vOTUs with marine viruses. vOTU_07 demonstrates protein-level homology with two Nonlabens phages. tBLASTx analysis of vOTU_35 revealed protein-level homology to two marine cyanophages. **(b)** Sequence alignment of vOTUs from extremophilic hosts. vOTU_17 demonstrate tBLASTx identity with sequences of two archaeal viruses. Sequence alignment of vOTU_39 shows tBLASTx identity with a high genomic GC content containing actinobacteriophage OscarSo (Genbank, OP434449.1) infecting *Microbacterium radiodurans*. **(c)** Viral proteome clustering of all vOTUs with Virclust indicates eight different clades (clusters 1, 2, 6, 10–12, 14, and 20) out of the predicted 22.

Protein-based clustering using vConTACT2 showed similarities of some South Georgia vOTUs to known marine phages. vOTU_07 shared similarities with Nonlabens phage P12024L (GenBank, JQ823123, Figures 4a,b), which originates from coastal seawater (Kang et al., 2012). vOTU_36 and vOTU_37, detected in rainwater, formed VC_747_0 sharing protein similarities with the Baltic Sea derived Flavobacterium phage vB_FspP_elemoA_8-9C (GenBank, MT497073). Based on VipTree, vOTU_35 was related to the widespread oceanic Cyanophage S-TIM5 (GenBank, NC_019516) (Sabehi et al., 2012; Baran et al., 2022) and estuarine Synechococcus phage S-CBWM1 (GenBank, NC_048106, Figure 5a) (Xu et al., 2018), vOTU_28, vOTU_29, vOTU_36, and vOTU_37 were related to different Flavobacterium phages of the genera *Elemovirus* and *Immutovirus* from the Baltic Sea (Hoetzinger et al., 2021), and vOTU_27 to different Cellulophaga phages isolated from the Baltic Sea (Holmfeldt et al., 2013) and North Sea (Bartlau et al., 2022). In total, 15 vOTUs were related to viruses known to have marine hosts or marine origin themselves (Figure 4a and Supplementary Table S4), providing evidence that the airborne viral community sampled here in South Georgia was influenced by marine input.

Functional annotations of the 39 vOTUs are given in Supplementary Table S5. vOTU_07, related to Nonlabens phages P12024S (GenBank, NC_018271) and P12024L (GenBank, NC_018272) (Figure 5a), had the most ORFs (19), encoding for proteins involved in host lytic machinery such as holin (ORF6) and peptidase (ORF9) (Shah et al., 2023). Sequence alignment and tBLASTx analysis of the vOTUs indicated potential similarities with viruses from different ecosystems. Several protein coding genes of vOTU_07 showed ~50% amino acid–level sequence identity with phages P12024S (GenBank, NC_018271) and P12024L (GenBank, NC_018272) (Figure 5a), which infect the marine bacterial host *Persicivirga* (family Flavobacteriaceae) (Kang et al., 2012). Additionally, vOTU_17 and vOTU_39 displayed protein similarities with viruses that infect extremophilic hosts, such as haloviruses HHTV-2 (GenBank, NC_021340.1) and HCTV-2 (GenBank, NC_021319.1), which infect the archaeon *Haloarcula californiae* isolated from salt brines (DSMZ, DSM 8905) (Sencilo et al., 2013) and phage OscarSo (GenBank, NC_073466.1), which infects *Microbacterium radiodurans* NRRL B-24799, a highly UV-tolerant bacterium (Zhang et al., 2010), respectively (Figure 5b).

Hierarchical and phylogenetic clustering of viral core proteins highlighted clustering into eight distinct clades (clusters 1, 2, 6, 10–12, 14 and 20) out of the predicted 22 (Figure 5c and Supplementary Table S6). These clusters in the hierarchical tree demonstrated protein overlaps and provided similar phylogenetic reconstructions as observed in the ViPtree analysis (Figure 4a). Clusters 1, 2, 6, and 11 had the greatest number of members (four), while cluster 20, comprised of just two members, shared the greatest number of common core genes. This cluster is formed from vOTU_36 and vOTU_37 and was related to Flavobacterium phage vB_FspP_elemoA_8-9C (GenBank, MT497073) in vConTACT2. Overall, we found high diversity of the proteome in the analyzed vOTUs, with proteins overlapping among scaffolds being phylogenetically similar.

### 3.3 Spatial distribution of selected protein homologs in the major oceans

**Figure 6:**
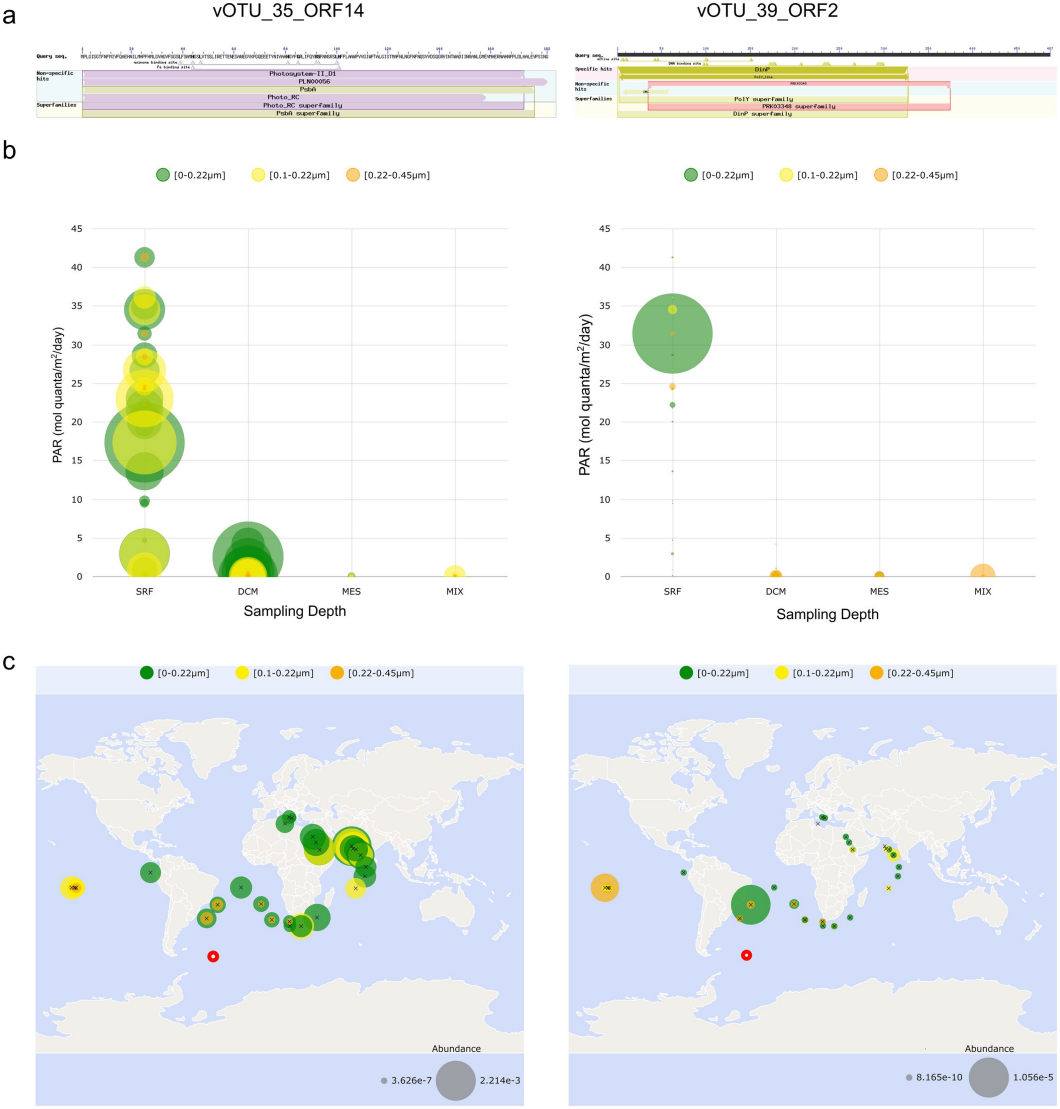
Distribution and abundance of viral protein sequence homologs in the major oceans. **(a)** Domain analysis of photosynthetic reaction center protein D1 derived from vOTU_35 and impB/mucB/samB derived from vOTU_39. **(b)** The abundance of the protein homolog for the photosynthetic reaction center protein D1 and impB/mucB/samB with a DinP domain in three different filtered fractions and across different sampling depths (SRF = surface water, DCM = deep chlorophyll maximum, MES = mesopelagic zone, MIX = mixed layer) and in relation to photosynthetically active radiation (PAR). **(c)** Distribution of protein homologs across stations of the Tara Ocean expedition based on BLASTp to the Tara Oceans Microbiome Reference Genome Catalog v1 OM-RGC_v1 as obtained from Ocean Gene Atlas. Red circle indicates the position of South Georgia. Size of bubble indicates abundance in **(b,c)**.

To investigate the ecological significance and environmental distribution of viral proteins with potential functional roles in photosynthesis and UV protection, we analyzed homologous sequences from vOTU_35 and vOTU_39 using a metagenomic database from the Tara Oceans project. VOTU_35 shared protein identity with the Cyanophage S-TIM5 (Figure 5a), which encoded the photosynthetic reaction center protein D1 with a psbA domain (vOTU_35_ORF14) (Figure 6a). The D1 and D2 reaction center proteins form a heterodimer responsible for the establishment of photosystem II in cyanobacteria (Kamiya and Shen, 2003; Shi and Schroder, 2004), which is the most light-sensitive complex of cyanobacteria (Pathak et al., 2019). Another protein from vOTU_39 shared similarity with a protein from the phage OscarSo (GenBank, NC_073466.1) that infects the extremophilic UV-tolerant host *Microbacterium radiodurans* (Zhang et al., 2010). The phage carries a gene encoding for the protein from the impB/mucB/samB family with a DinP domain having functions in the bacterial SOS (DNA damage) response and thus has relevance in UV protection (Pfam database, PF00817) (Smith and Walker, 1998) (Figure 6a). To understand the distribution of these protein sequences and the possible origin of the encoding vOTUs, we explored the biogeography of the protein sequences based on matches with the Ocean Gene Atlas. The global distribution of the two protein homologs shows distinct patterns in terms of geographic spread and relative abundance. The protein homolog of the photosynthetic reaction center protein D1 is widely distributed across Tara Oceans stations, with notable concentrations across the South Atlantic Ocean, western Indian Ocean, and eastern tropical Atlantic, as well as in parts of the central South Pacific. The highest abundances are observed in central Africa and the Indian Ocean region. This homolog appears across multiple size fractions, including 0–0.22 μm, 0.1–0.22 μm, and 0.22–0.45 μm, and exhibits a relatively high abundance range from 9.94e-6 to 1.02e-3. In contrast, the protein homolog impB/mucB/samB with a DinP domain has a more restricted distribution, being primarily detected across the South Atlantic Ocean, the western Indian Ocean, and the eastern equatorial Pacific Ocean. It is present in the same particle size fractions as the other homolog but at much lower abundances, ranging from 6.68e-9 to 1.06e-5. The highest abundances of this homolog are localized to a single prominent site in the South Atlantic Ocean (station Tara_076).

Homologs of photosynthetic reaction center protein D1 (vOTU_35_ORF14) were widely detected in several Tara Oceans sampling locations in the surface water layer (SRF) at varying PAR ranging from approximately 0.07 mol quanta m^−2^ day^−1^ to 41.3 mol quanta m^−2^ day^−1^ as compared to the mesopelagic zone (MES), where almost no PAR was observed, deep chlorophyll maximum (DCM) with PAR ranging from approximately 0.038 mol quanta m^−2^ day^−1^ to 2.52 mol quanta m^−2^ day^−1^, and the marine epipelagic mixed layer (MIX) with PAR ranging from approximately 0.00002 mol quanta m^−2^ day^−1^ to 0.000059 mol quanta m^−2^ day^−1^ (Figure 6b and Supplementary Table S7). A total of 2,625 hits were obtained for this protein, with an abundance value of 192,236. Taxonomic distribution of these homologs indicated a high prevalence of this protein in viral genomes (37%) (Supplementary Figure S1). The UV protection-related protein (vOTU_39_ORF2) was mostly abundant in the SRF as compared to the MES, DCM, and MIX layers; however, the distribution across the sampling locations was rather sparse as compared to vOTU_35_ORF14 (Figure 6b and Supplementary Tables S7, S8). A total of only 59 hits were obtained for this impB/mucB/samB family protein, and the number of abundance measures was 1924, while for the photosynthetic reaction center protein D1, we received 2,625 hits and the number of abundance measures was 192,236. Viral homologs of the impB/mucB/samB family protein could not be found. The closest homolog predicted by the Ocean Gene Atlas for this protein was the impB/mucB/samB and DinP domain-containing protein of the bacterium, *Vulcanimicrobium alpinum* (GenBank, WP_317995911.1) (Yabe et al., 2022), isolated from a fumarole ice cave at high altitude on the volcano Mount Erebus in Victoria Land, Antarctica (Supplementary Figure S2). Both protein homologs were present at sampling location TARA_076, closer to the sampling site of our study than most other Tara Ocean stations (Figure 6c).

### 3.4 Microbial community composition and host assignments

Only 11 hosts could be predicted for the 39 vOTUs using iPhoP. The vOTU_12–15 were assigned to *Haemophilus D parainfluenzae (confidence score 95.4),* vOTU_24 to *Pseudomonas* sp. (93.3), vOTU_26 to *Aquimarina brevivitae* (91.1), vOTU_28 to UBA1924 within the order *Phycisphaerales,* vOTU_30 to an unknown species of *Labilibaculum* (92.1), including high scores for *Labilibaculum filiforme* (90.8) and *Labilibaculum antarcticum* (90.1), and vOTU_37 to *Psychromonas* sp013619145 (91.4). *Aquimarina brevivitae, Labilibaculum filiforme,* and *Labilibaculum antarcticum* are bacteria previously found in marine sediments, and the latter two are psychrotolerant (Yoon et al., 2006; Vandieken et al., 2017; Watanabe et al., 2020). Microbial community profiling (mainly prokaryotes, with a small number of reads of Basidiomycota) showed that the airborne community was dominated by Actinobacteria, Proteobacteria (now Pseudomonadota) and unassigned taxa. More unassigned taxa were found in the inland DMP samples (up to 80% relative abundance) than at the coastal KEP samples (up to 18% relative abundance, Supplementary Table S9 and Supplementary Figure S3). To provide taxonomic and ecological context, identify potential host associations, and compare our sequences to previously reported viral populations, we performed BLAST searches of vOTUs against the IMG/VR database. This approach revealed hits to orders Ortervirales (vOTU_01, vOTU_23, vOTU_24) and Algavirales (vOTU_03), to the class Caudoviricetes (vOTU_07, vOTU_12 – vOTU_16, vOTU_18, vOTU_19, vOTU_20, vOTU_25 – vOTU_29, vOTU_33, vOTU_34 – vOTU_37, vOTU_39), and to the order Priklausovirales (vOTU_31). We acknowledge, however, that the distinction between viruses and retroelements can be challenging for Ortervirales-like sequences. Predicted hosts were of the phyla Actinobacteriota (vOTU_12 – vOTU_15), Alphaproteobacteria (vOTU_39), Saprospiraceae (vOTU_36 and vOTU_37), and Spirochaetota (vOTU_12 – vOTU_15). Most viral hits were to marine, aquatic or, seawater ecosystems from various parts of the world (Supplementary Table S10).

## 4 Discussion

### 4.1 Aerosolization of different viral groups, site effects, and biogeographic distribution

Our data show that the airborne viral community composition over South Georgia is influenced by the sampling site on a local scale (coastal vs. inland) and the sampling device used. We found typical eukaryotic viral groups like NCLDV, which are often parasitized by smaller DNA viruses, known informally as virophages. Both virus types have been previously reported after sampling from 15 m depth in Marguerite Bay in the Southern Ocean close to the Antarctic continent (Piedade et al., 2024) and to influence algal host-virus interactions in a meromictic Antarctic lake (Yau et al., 2011). About half of all the vOTUs identified corresponded to phages. Our data confirm that viruses of various groups become airborne and form the first baseline data on airborne viral communities from South Georgia, along with evidence of day-to-day fluctuations and of influence by the marine environment.

More vOTUs were assembled from and were present based on read mapping at the immediately coastal KEP site, suggesting aerosolization of local marine viruses. Several vOTUs shared proteins with known cyanophages, and phage isolates infecting typical marine heterotrophic bacteria and, additionally, typical marine prokaryotes such as Thaumarchaeota (*Nitrosopumilus* sp.), were part of the microbial community (Supplementary Figure S3). An earlier study in the Arctic explored viral distribution and adaptation at the air-sea interface and showed that virus dispersal across the Arctic might be facilitated by aerosolization of viruses residing in the sea-surface microlayer, the uppermost 1 mm surface layer in contact with the atmosphere (Rahlff et al., 2024). Viruses are active in the Antarctic microlayer (Vaqué et al., 2021) from where they can be emitted into aerosols (Aller et al., 2005). As our sampling sites were located on a remote Southern Ocean island far from other terrestrial influences, finding such small-scale local differences in marine contributions to the airborne viral community was not our initial expectation as, in absolute terms, both sites are physically close to the coast. High GC content vOTUs known to be present in air and rain samples from Swedish air (Rahlff et al., 2023) were detected on one sampling day in the dataset explored here. This was a day with particularly low wind speeds, sunny weather, and a wind direction of 238 degrees (west-south-west), the latter contrasting with all other sampling, where the wind direction was north-west to north–north-west (Supplementary Table S1). These high GC viruses shared protein identities with Microbacterium phage OscarSo (37.5% identity in the case of vOTU_39), which has a GC content of 69.2% (https://phagesdb.org/phages/OscarSo/), and with two archaeal viruses of the halophilic archaeon *Haloarcula* (e.g., 34.4 and 34.7% in the case of vOTU_17). OscarSo has been isolated from *Microbacterium radiodurans*, which is highly resistant to UV radiation and originates from sand of the Gobi Desert (Zhang et al., 2010). Hence, our findings provide some evidence that airborne viruses with high GC content could have extremophilic hosts, allowing speculation that such viruses have a non-marine origin and stem from the higher atmosphere or terrestrial influences such as desert dust. The majority of marine prokaryote genomes have a GC content ranging between 30 and 50% (Luo et al., 2015; Yan et al., 2021), and associated viruses typically mirror the GC content of their hosts (Bahir et al., 2009; Simon et al., 2021).

The distance between the two sampling sites, DMP and KEP, is approximately 2 km and, hence, our data overall confirm short-range dispersal of Antarctic viruses with bioaerosols, with likely influences from sea spray and wet precipitation as precipitation occurred during some of the sampling events (Supplementary Table S1). Viral alpha diversity did not differ significantly between the two sites, whereas bacterial diversity showed a significant difference (Malard et al., 2025). The marine origin of certain viruses and the spread of specific genes suggest that viruses are primarily sourced through atmospheric transfer over long distances. We speculate that viral communities are less influenced by a study site than bacteria, because of longer residence times and lower deposition velocities in air, since they are smaller (Reche et al., 2018; Alsante et al., 2021). Therefore, they can be dispersed further and can become part of another remote community more easily.

Proteomic alignment and classification of the vOTUs were consistent with the results of the phylogenetic and clustering analyses. We found sequence identity at the amino acid level of some vOTUs with viruses of typical marine bacteria and archaea. Specifically, two different proteins encoded by vOTU_35_ORF14 and vOTU_39_ORF2 shared several homologs that are widely distributed in the surface water layer of the major oceans (Pacific, Atlantic, and Indian Ocean), supporting a probable marine origin. While speculative, the functionality of these proteins and their potential involvement in modulating a host’s metabolism may be important. Phages present in diverse ecological habitats must be highly adaptive; for instance, this could involve the placement of structural genes in regions of above-average GC content of the genome (Das and Rahlff, 2024), encoding specialized proteins promoting host survival and extremotolerance, or reverting to lysogenic or pseudolysogenic life cycles (Hwang et al., 2021; Irby and Broddrick, 2024). Typical traits of marine phages include augmentation of host metabolism or enhancing viral fitness by the acquisition and expression of auxiliary metabolic genes (AMGs) (Heyerhoff et al., 2022). Considering that phage-derived psbA has been demonstrated to support host photosynthetic activity in surface water (Sharon et al., 2007), and the potential of the impB/mucB/samB gene cluster to support microbial growth in harsh environments is known (Wang et al., 2022), we hypothesize that vOTU_35_ORF14 and vOTU_39_ORF2 could potentially be AMGs supporting the replication of these viruses in the marine and atmospheric environments, respectively. This could be appropriate and adaptive, as organisms in both environments face challenging conditions, for instance, surface water in the open ocean is oligotrophic (Goldman, 1984), and the atmosphere is prone to desiccation and solar and UV radiation effects (Polymenakou, 2012).

### 4.2 Methodological challenges of sampling air viruses for metagenomics

Several viruses remained unclustered by vConTACT2 or could only be clustered with other viruses obtained in this study, suggesting the presence of previously unknown viral diversity. In interpreting our results, it is important to acknowledge that many viruses remain unclassified and undocumented in public repositories. As a result, the vConTACT2 reference database used in this study may not fully capture the diversity of viral sequences present in our samples, potentially leading to the underrepresentation of novel or poorly characterized viral taxa. In addition, the overall viral biomass in these air samples was low, a common challenge in aeromicrobiology (Harnpicharnchai et al., 2023; Hou et al., 2023). As in a previous air virus study (Jiang et al., 2025), this led us to lower the generally-applied 10 kb length cut-off for the analysis of vOTUs proposed by the standard viromics guidelines (Roux et al., 2017) to accept smaller, 5 kb, viral scaffolds to be used for viral presence/absence determination. Such a step might also be appropriate for studies in other low and ultra-low biomass systems, such as clean rooms or space equipment (Zhang et al., 2018; Highlander et al., 2023). However, accepting smaller fragments can also increase uncertainties in various downstream predictions as well as the probability of false-positive predictions. Because airborne viruses are present in low abundance, the detection of a vOTU in a sample is more likely to be affected by limited coverage compared to high biomass ecosystems, making it difficult to accurately interpret overlapping communities and day-to-day variation in diversity.

The type of sampling device used here clearly influenced the viral findings. The dry Coriolis sampled few viruses (a single virus was assembled) and none were found by mapping reads from dry Coriolis samples to vOTUs retrieved from other samplers. According to the manufacturer’s information, the Coriolis Compact air sampler has a flow rate of 50 L min^−1^ and is described as a device capable of virus sampling, but this device is not mentioned in the academic literature on this subject. The wet Coriolis (Coriolis *μ*) has been used extensively for viral air sampling (Verreault et al., 2011; Hostyn et al., 2025; Letourneau et al., 2025). It has a higher flow rate of up to 300 L min^−1^ and collects particles into a liquid and, thus, might be more efficient than the dry Coriolis. Additionally, the liquid might be more suitable for conserving the viral particles. Both Coriolis samplers have a size range of 500 nm – 10 μm for collected particles, which should exclude most free and small viruses. As we have no information on the particle size of the sampled viruses here, we can only speculate whether small viruses were not sampled or were only collected if attached to larger particles. Further tests are required to thoroughly validate the devices used for viral sampling, including tests on the stability of viral nucleic acids on filters and in liquid, as degradation of nucleic acids is possible during long-term air sampling (Boifot et al., 2024). Instead of sampling on 0.2 μm pore-sized membranes or Sterivex filters, future studies of viruses collected into liquid could be iron-flocculated or subjected to ultrafiltration and processed as viromes, as done for aquatic samples (Langenfeld et al., 2021).

A larger diversity of viruses was sampled with the rain collector. Rain and aerosol-derived viruses can be genomically very different (Rahlff et al., 2023), while airborne microbial communities also vary between precipitation types and with seasonal variations in Antarctica (Els et al., 2019; Malard et al., 2022). Work by Jiang et al. (2025) supports that the atmosphere harbors a distinct viral community while also revealing habitat-specific viral assemblages and AMGs. Supporting previous findings (Rahlff et al., 2023), our work revealed VCs only found in the rainwater samples, which could indicate that they originate from a higher altitude. The collection of different, non-overlapping vOTUs by our two sampling devices suggests that using a funnel collector alongside an impinger air sampling system provides a more comprehensive representation of the airborne viral community, which can vary between aerosols and rain.

## Data Availability

Sequencing data are stored in NCBI’s SRA under the Bioproject PRJNA1107129, Biosample IDs: SAMN41160173 - SAMN41160182. The vOTUs are deposited at figshare under doi: 10.6084/m9.figshare.26536105 and in NCBI’s BankIt under accession numbers PV563644 - PV563682. Weather data are downloadable via https://legacy.bas.ac.uk/cgi-bin/metdb-form-2.pl?tabletouse=U_MET.GRYTVIKEN_AWS&complex=1&idmask=.....&acct=u_met&pass=weather.
