# Supplementary material for: Diversity of DNA viruses in the atmosphere of sub-Antarctic South Georgia
Source: Front Microbiol. 2026 Jan 28;16:1726848. doi: 10.3389/fmicb.2025.1726848 (PMC12893351; doi:10.3389/fmicb.2025.1726848)
Supplement: Supplementary file 1 [file Data_Sheet_1.PDF]

Diversity of DNA Viruses in the Atmosphere of Sub-Antarctic South Georgia

Ritam Das[1,2,3], Lucie Malard[4,5], David A. Pearce[6,7], Peter Convey[7,8,9,10,11], Janina Rahlff[1,12,13,14#]

[1]Aero-Aquatic Virus Research Group, Faculty of Mathematics and Computer Science, Friedrich Schiller University Jena, Jena, Germany

[2]International Max Planck Research School for Biology and Computation (IMPRS-BAC), Max Planck Institute for Molecular Genetics, Berlin, Germany

[3]Department of Biology, Chemistry, Pharmacy, Freie Universität Berlin, Berlin, Germany

[4]Department F.-A. Forel for Environmental and Aquatic Sciences, University of Geneva, Geneva, Switzerland

[5]Department of Ecology and Evolution, University of Lausanne, Lausanne, Switzerland

[6]Department of Applied Sciences, Faculty of Health and Life Sciences, Northumbria University, Newcastle-upon-Tyne, United Kingdom

[7]British Antarctic Survey, Natural Environment Research Council, Cambridge, United Kingdom

[8]Department of Applied Sciences, Zoology, University of Johannesburg, Auckland Park, South Africa

[9]Millennium Institute – Biodiversity of Antarctic and Sub-Antarctic Ecosystems (BASE), Santiago, Chile

[10]Cape Horn International Center, Puerto Williams, Chile

[11]School of Biosciences, University of Birmingham, Edgbaston, United Kingdom

[12]Leibniz Institute on Aging - Fritz Lipmann Institute (FLI), Jena, Germany

[13]European Virus Bioinformatics Center, Jena, Germany

[14]Centre for Ecology and Evolution in Microbial Model Systems (EEMiS), Department of Biology and Environmental Science, Linnaeus University, Kalmar, Sweden

#corresponding author: Janina.rahlff@lnu.se

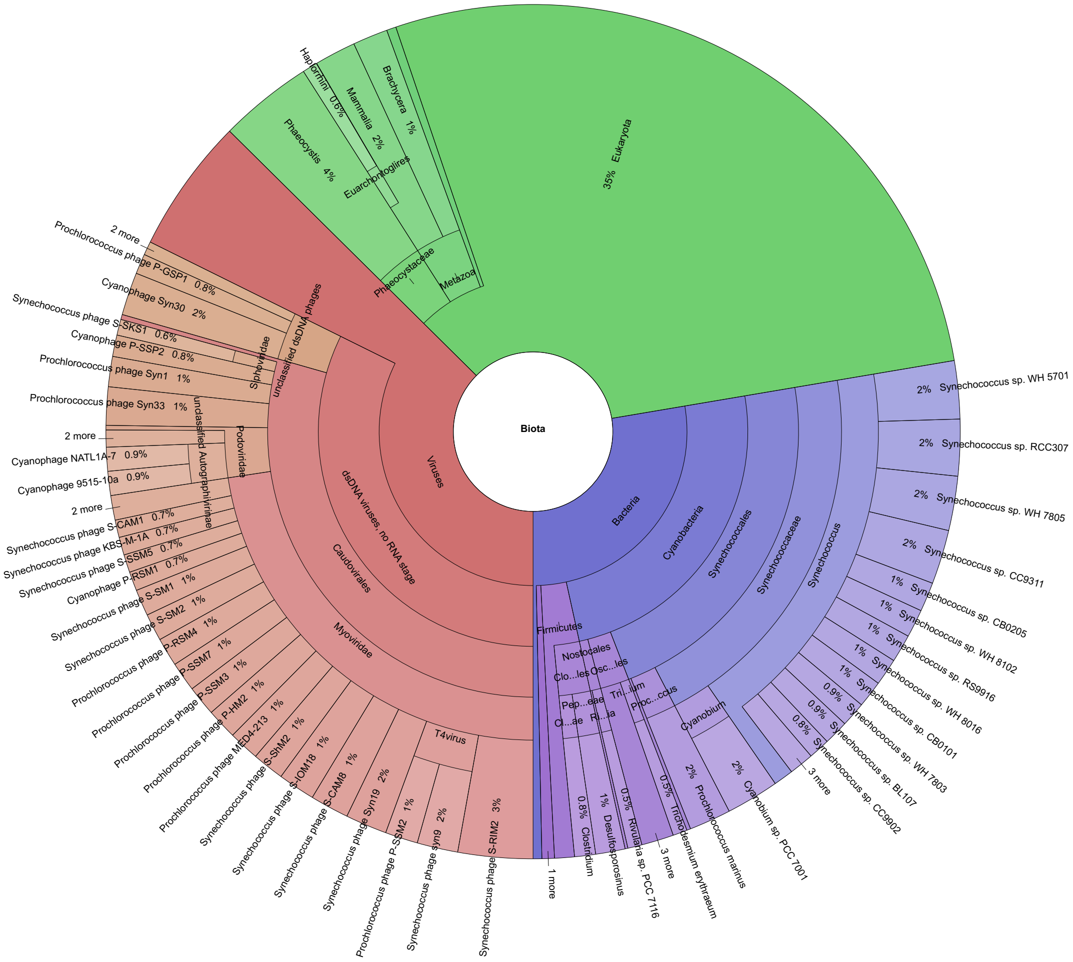

Figure S1: Protein homolog analysis of Photosynthetic reaction centre protein D1 (psbA) vOTU_35_ORF2. Krona plot demonstrates the proportion of the homologs in viral genome isolates from the Tara Oceans Microbiome Reference Genome Catalog v1 OM-RGC_v1.

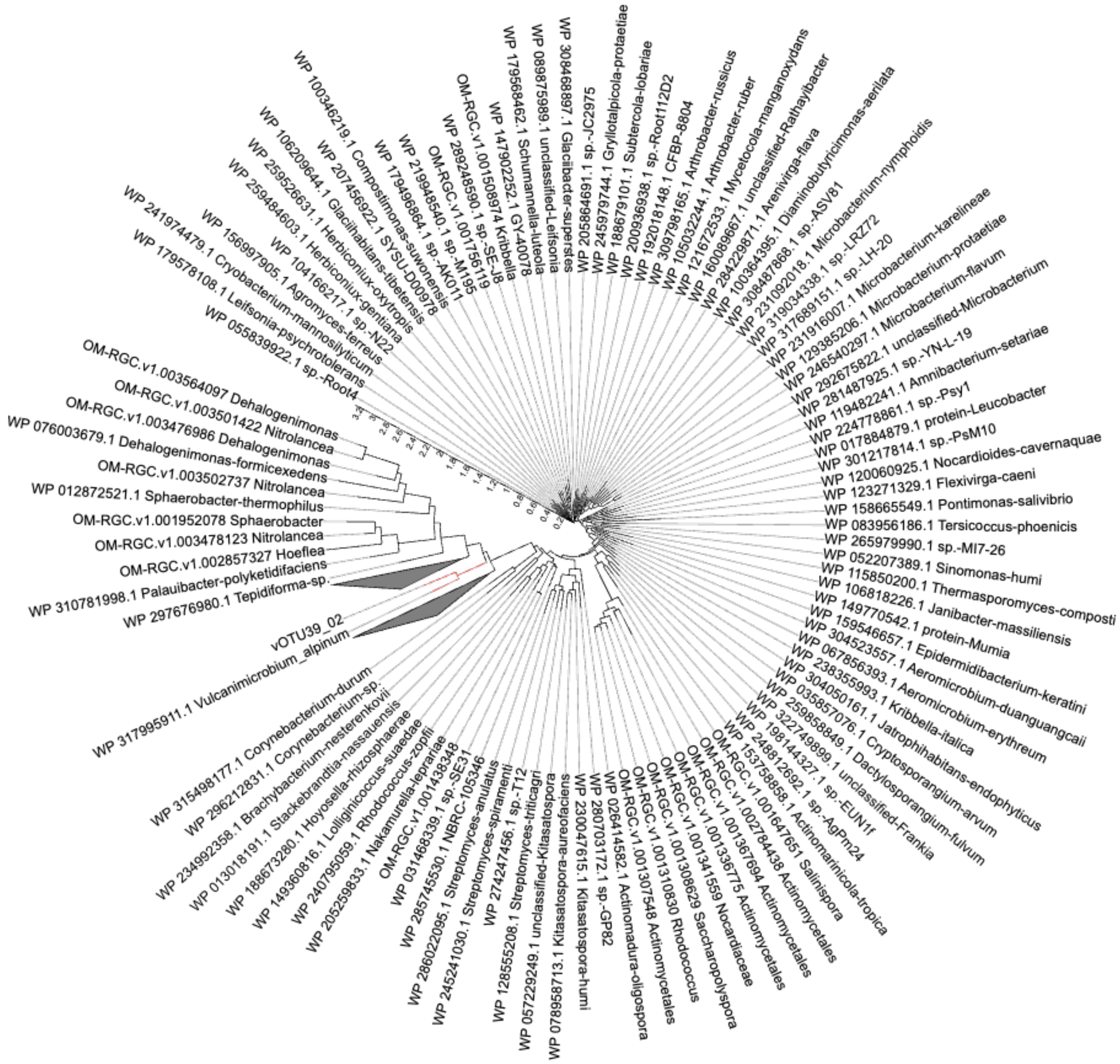

Figure S2: Protein homolog analysis of impB/mucB/samB from vOTU_39. Phylogeny of impB/mucB/samB from vOTU_39_02 showing close similarity and ancestry with the impB/mucB/samB with a DinP domain containing protein of the extremophile *Vulcanimicrobium alpinum* (edges highlighted in red). Black triangles indicate collapsed nodes.

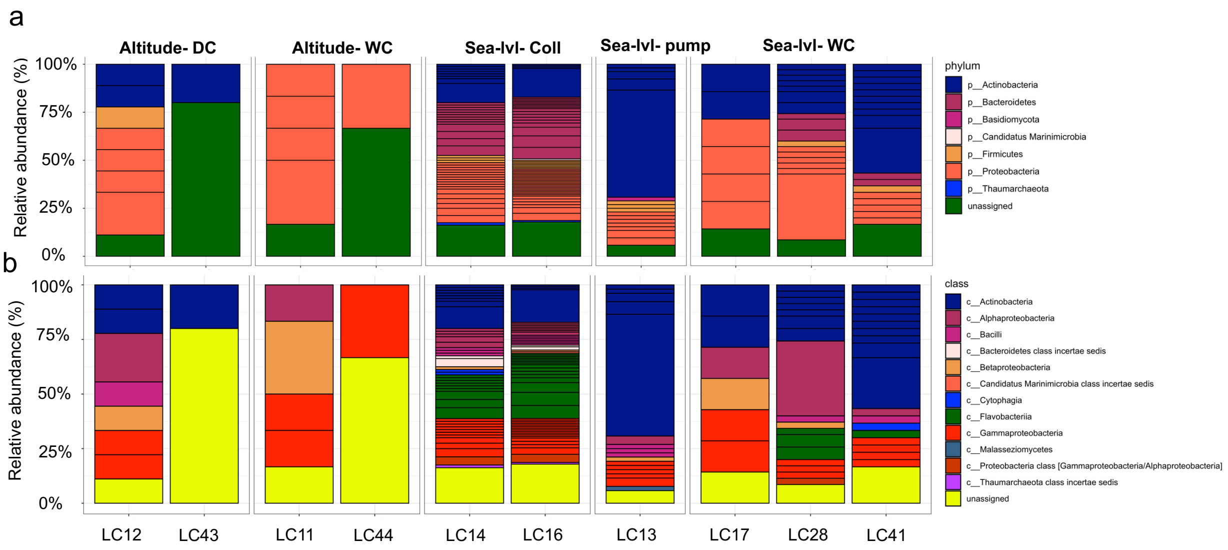

Figure S3: Relative abundance of air-derived prokaryotes based on a) phylum and b) class. DC=dry Coriolis, Sea-lvl= sea level, WC=wet Coriolis. LC refers to an internal sample number.
